# Supporting Role of Society and Firms to COVID-19 Management among Medical Practitioners

**DOI:** 10.3390/ijerph17217961

**Published:** 2020-10-29

**Authors:** GholamReza Zandi, Imran Shahzad, Muhammad Farrukh, Sebastian Kot

**Affiliations:** 1 Universiti Kuala Lumpur Business School, 54000 Kuala Lumpur, Malaysia; zandi@unikl.edu.my; 2 Post Graduate Center, Limkokwing University of Creative Technology, Cyberjaya 63000, Malaysia; 3 School of Management and Economics, Beijing Institute of Technology, Beijing 100080, China; mfarrukhiqbal@hotmail.com; 4 Faculty of Management Sciences, Department of Business Administration, Ilma University, Karachi 72400, Pakistan; 5 The Management Faculty, Częstochowa University of Technology, 42-2001 Częstochowa, Poland; 6 Faculty of Economics and Management Sciences, North-West University, Vaal Triangle Campus, P.O. Box 1174, Vanderbijlpark 1900, South Africa

**Keywords:** COVID-19, job stress, exposure, organizational commitment, quarantine, social support, perceived organizational support

## Abstract

Measurement of job stress and employees’ commitment are few of the admired topics in the corporate world amongst business writers. With a principal aim to trial the blow of exposure to COVID-19 patients on doctors’ job stress and commitment, in Pakistan; data have been collected through 7-10 min telephonic interview from voluntary participants and a sample of 129 responses were analyzed by Structure Equation Modeling-Partial Least Square (SEM-PLS) path modeling through Smart PLS 3.2. The results of the study indicated; direct positive & significant impact of Extent of Exposure on Job Stress while direct negative, significant association with Commitment. Job Stress also observed having direct negative impact on commitment. The Extent of Exposure-Job Stress relationship was also found stronger among group of doctors having Low level of Perceived Organizational Support and weaker among group of doctors having High level of Perceive Organizational support. Perceived Organizational Support showed a moderating effect on the Extent of Exposure-Job Stress relationship; while, Social Support showed no moderation. Researchers are required to investigate more and management of the medical services providers (both hospitals and government) needs to focus on doctors’ perception about Organizational Support, as doctors show no concern about the support from society as long as their well-being is cared for by respective hospitals. This study is an effort to stimulate more empirical evidence towards the treating and handling of COVID-19 patients and the psychological well-being of doctors.

## 1. Introduction

In Pakistan, the number of patients with COVID-19 has dramatically increased since 26 February 2020, when the first patient of COVID-19 was identified. Therefore, the main objective of the current study was to examine the stress that doctors feel about their own health situation during their interaction with COVID-19 patients in Pakistan. As per the theory of reasoned action, it is generally thought that Job Stress (JS) would be mounting along with health workers, specifically among doctors, because of their interaction with COVID-19 patients, as one of the few main possible reasons. According to current reports, Job Stress (JS) can be an outcome of both interpersonal and intrapersonal aspects, such as a poor work environment, severe family conditions, and fear of the risk of accidental infection during the interaction process, and inappropriate personal coping strategies that can cause chronic stress.

Health workers normally deal with patients with chronic diseases, serious illness, and they are accustomed to their job tasks, but treatment of COVID-19 patients may be stressful, in particular. Initially, COVID-19 was devised as a respiratory lung-related viral infection that increases the chances of accidental infection through near contact with patients and it is further thought to be considerably more highly probable to catch than was originally thought [1,2]. Alternatively, doctors may view caring for COVID-19 patients as a positive experience, for example, high levels of personal satisfaction, positive feelings and achievement. Here, some researchers reported that doctors have taken it as a challenge, are found more committed than ever before and have performed long-hour duties in hospitals to treat COVID-19 patients [3] which shows doctors’ commitment with work, hospitals, society and humanity. Extent of Exposure (EE) to COVID-19 patients is positively related to job stress at work and Extent of Exposure (EE) of doctors with COVID-19 patients can positively influence Organizational Commitment (OC) of doctors with their profession and job. In this study, we did not expect such feelings to be widespread for several reasons, as given below:

Firstly, reactions to the COVID-19 pandemic and individuals infected have been observed at an extreme level [3]. Some of the researchers suggested that these reactions are part of the COVID-19-related viral infection that stems from the nature of the illness and its association with groups already stigmatized before the widespread of COVID-19 in Pakistan [4].

Secondly, Khan, Maqsood, Hussain and Zeeshan [5] pointed out that for the various physical and psychological symptoms of COVID-19, the protective procedures that medical staff must follow when caring for COVID-19 patients, as well as the negative reactions to COVID-19 in public at large, may cause exposure to COVID-19 patients to be particularly stressful. Moreover, the risk of accidental infection may cause health care providers to be fearful, anxious, or angry regarding such contacts. Although some of these negative reactions are similar to those engendered by contact with cancer patients [6], the fact that cancer generally is not infectious, whereas COVID-19 is, may cause such fears and their effects to be much stronger.

Finally, results from several sources suggest that health care providers are indeed experiencing Job Stress (JS) over their contact with COVID-19 patients. For example, some researchers from China reported that a sample of hospital workers viewed COVID-19 related care as one of the most stressful parts of their job that created stress and it quadrupled in Pakistan with a provoking claim of one of the prime new agencies “The Dawn”, on 29 April 2020, as it reported “Corona virus Infects 480 Pakistani Health Workers, Kills 3 Doctors”. Furthermore, when the World Health Organization officially named the disease as “Corona virus disease 2019”, even those considered as the best known and well informed appeared to find such contact highly stressful [7]. As reported by Rana, Mukhtar, and Mukhtar [8], the “World Health Organization” (WHO) on January 30, 2020, declared the occurrence of the novel corona virus and declared a PHEIC (Public Health Emergency of International Concern), which is the sixth PHEIC under the IHR (International Health Regulations) after H1N1 Influenza (2009), Polio (2014), Ebola in West Africa (2014), Zika (2016), Ebola in Democratic Republic of Congo DRC (2019) (Euro-surveillance Editorial, 2020). The COVID-19 outbreak seems to be being handled poorly around the world (especially on social media) and appears more stressful and fearful as compared to the other six PHEICs announced by the WHO because of: (i) worldwide quarantine (ii) the point of transmission, (iii) speed of outbreak, (iv) fear of getting stigmatized, and (v) most of the researchers reported earlier that news on social media provokes alarm in a very unmannered way which creates distress and reduces job commitment of health workers, by announcement of the first case of suicides in India by Goyal, Chauhan, Chhikara, Gupta, and Singh [9].

### 1.1. Relationship between Extent of Exposure (EE) and Job Stress (JS)

The spread is wide and devastating for the developed economies; in this situation, developing countries like Pakistan, despite having a number of well-informed medical professionals, the persistent spread of the virus may be one of the primary reasons for distress. International news and reports also proved COVID-19 to be one of the main reasons for the stress as “The pandemic not only brought the high mortality rate from the viral infection but also psychological rest and mental catastrophe to the rest of the world” [10], frustration, helplessness, and adjustment challenges [11]. Job Stress (JS) itself has demonstrated severe consequences for the working environment and overall organization. In the past, most studies used Job Stress as a predictor of job satisfaction, employee performance, organizational citizenship behavior, job burnout, organizational commitment, and intention to rotate [12,13,14].

On the other hand, few studies have introduced Job Stress (JS) to interfere with rotation intentions [6,7,8]. Furthermore, researchers believe that employees with a high level of Job Stress (JS) do not perform best, nor do they remain loyal to their work and organization, which ultimately increases the level of employees’ willingness to leave the firms. Heponiemi, Presseau and Elovainio [15,16] also support the use of Job Stress (JS) as an intervening variable with indirect impact instead of direct. Researchers believe that Job Stress (JS) has a minor role in the withdrawal process. Rana, Mukhtar, and Mukhtar [8] and Nawaz, K. et al. [17] further highlighted that “COVID-19” has a considerably high potential for psychological fear contagion as well and could result in a prevalent multitude of psychological problems such as fear, anxiety, stigma, prejudice, marginalization towards the disease. Further to this, worldwide quarantine practices at a significant level are expected to generate widespread hysteria, distress, anxiety, and much more specifically for the doctors and other medical-related staff. Based on the discussion, the hypothesis was developed as:

**Hypothesis** **1.** *Doctors’ Extent of Exposure (EE) with COVID-19 patients has a positive impact on Job Stress (JS) at work*.

### 1.2. Relationship among Extent of Exposure (EE), Job Stress (JS) and Organizational Commitment (OC)

Samson, D. et al. [18] pointed out that organizational commitment is a psychological state that characterizes the relationship between employees and their organizations and influences the decision to continue serving as a member of the organization. The three types of commitment highly used in literature are affective, continuous and normative commitment, where Affective Commitment (AC) refers to the attachment or identity of employees to their organization and tasks [19]. Continuity commitment refers to employees’ perceptions of the costs associated with leaving their organization [20]. Finally, the normative commitment points out the understanding of the employees of their organizational obligations [21]. Further, some of the researchers in their meta-analysis of 88 studies investigated the relationship between perceptual control and other work variables (such as job satisfaction, organizational commitment, emotional distress and absenteeism), and concluded with a number of factors that affect employee engagement with organization. “Employees’ satisfaction with the quality of the supervision and career growth opportunities found having direct impact on organizational commitment. Further variables that has significant relationship with employee commitment to organization were; employee empowerment, job-motivating potential, effective leadership, acceptance by co-workers, role ambiguity/clarity and conflict” [22].

Equity in reward distribution, role clarity, freedom from conflict and supervisor support, autonomy, fairness of performance-based rewards, opportunity for self expression, opportunity for advancement and participation in decision making are the determinants of organizational commitment [18]. Organizational commitment is found to have close association with other organizational aspects such as employee turnover intentions, distress, absenteeism from work, and work efforts. Manager subordinate relationship according to the Leader Member Exchange (LMX) theory: leaders can increase subordinates’ commitment with their organization [23]. Distress and interaction with patients of COVID-19 are two of the possible reasons that can effect workers’ commitment to their job and firm. Further to this, work-related stress plays a mediating role between inputs and employees’ outcomes [24].

Hence, for all of these reasons, we expected that the extent of doctors’ exposure to COVID-19 patients as part of their role would be positively associated with job stress at work and would tend to further generate a number of positive and negative outcomes related to their commitment to hospitals. These were a few of the concentrated factors selected for this study and based on the above discussed arguments, this study postulated:

**Hypothesis** **2.** *Doctors’ Extent of Exposure (EE) with COVID-19 patients has a negative impact on Organizational Commitment (OC)*.

**Hypothesis** **3.** *Doctors’ Job Stress (JS) has a negative impact on Organizational Commitment (OC)*.

**Hypothesis** **4.** *Job Stress (JS) mediates the relationship between doctors’ Extent of Exposure (EE) with COVID-19 patients and Organizational Commitment (OC)*.

These expectations are also consistent with substantial literature suggesting that, to the extent that caring for COVID-19 patients does result in heightened job stress and reduce commitment of doctors, it is important to identify what may lessen COVID-19-related stress and how stress and interaction with COVID-19 patients can affect the doctors’ commitment with respective organizations (hospital).

### 1.3. Relationship among Perceived Organizational Support (POS), Job Stress (JS) and Organizational Commitment (OC)

Further to this, this study considered two specific potential moderators of the relationships among Extent of Exposure (EE), Job Stress (JS), Organizational Commitment (OC), Perceived Organizational Support (POS) and Social Support (SS). Cohen [25] discussed four functions of support that help to protect individuals from deleterious effects of stressors. These functions of support and related posited effects could be considered as: First, support can actively promote a sense of self-esteem and acceptance by allowing people to know values and acceptance without being affected by problems or deficiencies. For example, feelings of helplessness and inferiority often accompany pain. Support can counteract this destructive thinking and increase personal awareness of one’s ability to cope with stress. Second, support can function as an information function and can help people interpret, understand, and respond to potential stressors in a functional way. Third, support can simply meet the needs of the company and social contact, which can lead to a “feeling of belonging” and help to distract people from the same stressors. Finally, support can play a role in providing people with the material resources and services necessary to help in overcoming the possible stressors.

In this article, it has been shown how Perceived Organizational Support (POS) and Social Support (SS) may serve those diverse functions and hence help to ameliorate the effects of exposure to COVID-19 patients on Job Stress (JS). These beneficial effects of support operate through their influence on both appraisals of the stressor and resources and options for coping with it [26].

Perceived Organizational Support (POS) refers to the global belief that people value their contribution to the organizations that care about their well-being, listen to their complaints, that provide help (when workers encounter problems) and fair treatment [27,28]. Research has linked organizational support to positive organizational outcomes [27], but has not yet explored its role in work-related stress experiences. In this study, it is hoped that organizational support will facilitate the relationship between Extent of Exposure (EE) and Job Stress (JS). Organizational support is particularly relevant to maintaining and improving self-esteem and providing information, material and resources. Eisenberger, Huntington, Hutchison, and Sowa [27] proposed that a sense of organizational support can satisfy individual needs for dignity.

This function of organizational support may be particularly relevant to doctors’ exposure to COVID-19 patients. As was earlier noted, workers’ reactions to such exposure may include fear, resentment, and a desire to avoid the contact [29]. These reactions, in turn, can reduce the employee’s level of self-esteem and their perception of the employee’s ability to cope with stress, due to the employers’ concern for the employees about their work effort and well-being. This understanding of organizational support helps them to work hard to achieve organizational goals, develop organizational commitment, reduce turnover intentions, absenteeism, and more efforts into additional roles [27,30,31].

Doctors may view their own distressed reactions as unprofessional, not in keeping with their commitment to care for patients in general, and negatively reflecting on themselves. They may feel guilty about their own doubts and misgivings and potential reactions to COVID-19 patients and see these feelings as a sign of personal deficiencies. These threats to self-esteem can reduce their awareness of the ability to cope with exposure to COVID-19, thus exacerbating the problem. The recruiting organization is believed to genuinely value and care about any potential self-esteem that may threaten self-accusation and improve the overall level of self-esteem, increasing awareness and ability to cope with exposure to COVID-19 patients. When perceptions of organizational support are high, doctors are also likely to believe that their organization is providing them with all the relevant information regarding COVID-19, their personal risk of accidental infection, and what they can do to protect themselves, and to trust the accuracy of this information. These perceptions may lower levels of distress since they are likely to lessen the tendency to exaggerate perceptions of the risk of accidental infection, which results in heightened inappropriate appraisals of exposure. Having complete and accurate information is also likely to enhance perceptions of the extent of resources and options for coping since doctors will know what they need to do to minimize risk and will have more accurate perceptions regarding the extent to which the situation is under control.

Ultimately, gaining a high level of organizational support can lead clinicians to believe that organizations will provide them with the resources they need to cope with their source of stress, thereby reducing the distress caused by exposure. Such resources may include time off from work, the possibility of reassignment to another unit if stress levels become too great and counseling services. Knowing that these and other resources are available may enhance perceptions of resources and options for coping and lead to less experienced distress.

Thus, the information functions of organizational support are likely to result in doctors’ appraising exposure to COVID-19 patients mildly, and the esteem, information, and material resource functions are likely to contribute to doctors’ feeling better able to cope with this potential stressor. All in all, organizational support’s effects on both the appraisal of the stressor and resources and options for coping should diminish reactions to exposure to COVID-19 patients. Hence, for this study, the following has been postulated:

**Hypothesis** **5.** *Perceived Organizational Support (POS) moderates the relationship between Extent of Exposure (EE) to COVID-19 patients and Job Stress (JS). Further, this relationship is stronger with Low Perceived Organizational Support (LPOS) and weaker with High Perceived Organizational Support (HPOS)*.

### 1.4. Relationship among Social Support (SS), Job Stress (JS) and Organizational Commitment (OC)

Social support is generally defined as the existence and availability of other people that people can trust and let them know that they value and care about them. Although social support helps to promote psychological adjustment and general well-being, Chu, Saucier, and Hafner emphasized this through meta-analysis. It also may be important in mitigating the deleterious effects of particular stressors. More specifically, it has been suggested that social support protects people from some of the negative influences of stressful events and circumstances [32]. Flynn Kecmanovic, and Alloy [33] reviewed the literature relevant to this idea and found support for buffering effects of social support, especially in research in which the social support measures assessed interpersonal resources that are in response to an individual’s needs or are relevant to the experienced stressors. Further to this, Xiao, Zhang, Kong, Li, and Yang, [34] highlighted their recommendation that social support fulfills at least two important functions for doctors exposed to COVID-19 patients: esteem functions and social companionship functions.

When doctors have others to talk to and depend on, others who really care about them and accept them totally, the threats to self-esteem mentioned previously may be lessened. Harley, A. E. et al., [35] found that being able to talk about one’s fears and apprehensions to others who value you and do not think any less of you for your misgivings can cause self-esteem to increase. Doctors’ self-esteem may be maintained in light of the threat of COVID-19 exposure, and they may feel better able to cope with the stressors. The companionship functions of social support may also be important in mitigating the effects of the extent of exposure on job stress for at least two reasons. First, having others whom they can spend time with in social and leisure activities may help distract doctors from their concerns over contact with COVID-19 patients and may also help them to recover from the potential strain of exposure to those patients and the extra vigilance it may impose upon their work behaviors and routines. Second, as mentioned above, COVID-19 carries a respiratory viral and has provoked intensely negative reactions in the public at large. Doctors caring for patients with COVID-19 may actually feel infected with the virus due to professional contact with COVID-19 patients, and relationships with family and friends become strained due to such contact. This may occur because others may perceive a doctor in contact with COVID-19 patients as being at risk of infection and may even fear contacting the doctor. Such potential infection may result in medical staff’s appraising contact with COVID-19 patients as highly threatening and in their feeling unable to cope with this stressor.

The companionship functions of social support may result in doctors’ not experiencing infection themselves from their contact with COVID-19 patients. Hence, doctors may react less negatively to such contact and feel better able to cope with it. All in all, the companionship functions of social support are likely to result in doctors’ appraising exposure to COVID-19 patients less negatively than they would otherwise, and both the companionship and esteem functions are likely to result in doctors’ feeling better able to cope with this stressor; overall, COVID-19 related distress should thus be diminished. Arnberg, Hultman, Michel, and Lundin [36] suggested that social support moderates the stressor–distress relationship after disasters. Social support proved a moderator, which might help explain discrepant findings and point to refinements of post-disaster interventions. Hence, this study postulated:

**Hypothesis** **6.** *Social Support (SS) moderates the relationship between Extent of Exposure (EE) to COVID-19 patients and Job Stress (JS). Further, this relationship was stronger with Low Perceived Organizational Support (LPOS) and weaker with High Perceived Organizational Support (HPOS)*.

## 2. Materials and Methods

### 2.1. Process

**Figure 1:**
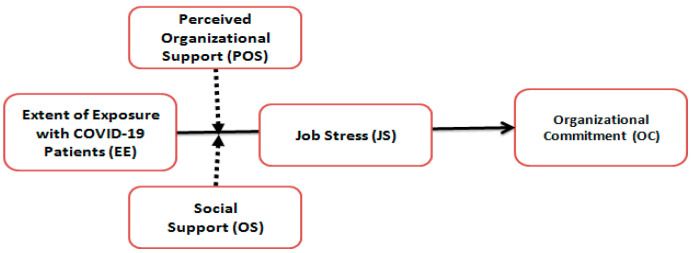
Conceptual framework.

Based on the developed hypothesis, the theoretical model is shown in Figure 1. In order to deal with a huge expected number of patients and maintain the social distance among patients and their relatives, the government of Pakistan, through the Ministry of National Health Services (MNHS), National Coordination Committee (NCC), National Security Committee (NSC) and Disaster Management Cell (DMC), managed eight quarantine centers covering the main cities while hundreds of hospitals were also converted to the quarantine centers around the country (voice of America, 28 March 2020). In the same way, doctors (who were members of the Pakistan Medical and Dental Council (PMDC) were appointed and relocated to perform roles and duties in most affected areas. As doctors were not uniformly appointed, this forced telephonic data collection, on structured questionnaires.

### 2.2. Participants

As doctors are not uniformly distributed across the entire country but rather are concentrated in big cities such as Karachi, Lahore, Islamabad, Faisalabad, Peshawar, Multan, Sukhar, Taftan border, convenient sampling was applied. The contact details of the doctors were obtained from the official website (www.covid.gov.pk) specifically designed by the government of Pakistan to cure the patients and block the spread of COVID-19. Doctors were contacted through given telephone numbers and in the first phase 349 doctors in eight big quarantine centers were asked to participate in the survey and have their responses recorded; this yielded eighty-four (84) responses. Secondly, 210 more contacts could generate forty-five (45) responses from the hospitals that were converted to the quarantine centers. All the doctors were those who were in direct interaction or exposure to COVID-19 patients. Participation was voluntary and responses were anonymous. The total contacted doctors were 559 while for this study only 129 responses were collected which depicted a 23% response rate. This was an exploratory study based on primary data collection through a questionnaire; therefore, a quantitative research method was applied. The questionnaire was divided into two portions: the first portion comprised of four demographical questions (age, gender, years of experience and marital status) while in the second portion, employees answered forty-two (42) questions covering five areas, Extent of Exposure (EE), Job Stress (JS), Organizational Commitment (OC), Social Support (SS) and Perceived Organizational Support (POS).

### 2.3. Measuring the Instrument

Extent of Exposure (EE) to COVID-19 patients was measured by three (03) items; the scale has been opted from the earlier work carried out for measuring the perceived extent of nurses’ exposure to HIV/AIDS patients [37]. The items composing the scale were as follows: “During the last three months approximately what percentage of your time at work did you spend caring for COVID-19 patients? During the last three months approximately what percentage of the patients you treated for having COVID-19?” and “To What extent does your work as a doctor exposed you to COVID-19 patients?” For the first two questions, respondents were asked to write down the relevant percentages. For the questions, doctors responded on a six-point scale ranging from 1—no extent to 6—great extent. In this study, responses were standardized to each of the three questions by converting them to z-scores prior to summing them to arrive at an overall score.

Job Stress (JS) has been calculated by a number of measures used in business and health sciences literature, e.g., General Health Questionnaire—GHQ [38], Occupational Stress Index—OSI [39], the General Wellbeing Questionnaire—GWQ [40], Perceived Stress Scale—PSS [25] and Subjective Well-being Inventory—SWI [41]. In this study, job stress was measured by an earlier tested scale, developed by Holmgren et al. (2009). It consists of twenty-one (21) questions. These questions are general, so there is relatively no content for specific subgroups. The questions on the Perceived Stress Scale refer to feelings and thoughts in the past three months. In each case, how often did the interviewee feel a certain way? The score of the Perceived Stress Scale 04 is obtained by inverting the scores of four positive items, e.g., 4, 5, 7 and 8 and then summing across all four (04) items which makes it suitable to use in a clinical setting without any specific diagnosis [42]—covering the interaction between personal and environmental factors [43], already used for work-related stress om a primary health care setting [44], tested for self-reported stress in the health industry, and equally used for both genders [43].

Perceived Organizational Support (POS) has been measured by using six (06) items covering six dimensions (employees’ concerns, employees’ goals and values, overall employee satisfaction, employees’ opinion, performance and their well-being) that were adapted from the Survey of Perceived Organizational Support (SPOS) in 1986 and 1990 by Eisenberger, Huntington, Hutchison, and Sowa, and Eisenberger, Fasolo, and LaMastro, repectively. Further to this, POS was measured on a six-point Likert scale where scores from 1 to 3 were considered as Low Perceived Organizational Support (LPOS) while scores from 4–6 were considered as High Perceived Organizational Support (HPOS).

Organizational Commitment (OC) was measured using six (06) research items found in the earlier work of Mowday, Porter and Steers [45], which covered: “official responsibilities, future of the organization, choice of the current organizations over other available options, organizational reputation, social impact and organizational culture and values”. Sample research items were “I am willing to put my effort beyond my official responsibility to help my hospital to be a successful in saving lives, I really care about the future of my firm and people; I feel proud to tell others that I am working with this hospital and location and I find my values and my hospital values are very similar.”

Social Support (SS) was measured with the satisfaction with the social support scale from six (06) items adopted from the short form of the Social Support Questionnaire (SSQ) earlier invented in 1983 by Sarason et al. where the sample items were: list the people whom they can turn to and rely on in a given set of circumstances and indicate how satisfied they are with the support available to them. Further to this; Social Support (SS) was measured on a six-point Likert scale, where scores from 1 to 3 were marked as Low Social Support (LSS) while scores from 4–6 were marked as High Social Support (HSS).

### 2.4. Data Analysis

The study used the Partial Least Square (PLS) Structural Equation Modelling (PLS-SEM) technique with the help of SmartPLS software to test the hypothesized relationships. This technique is suitable for the small sample size [46]. PLS-SEM has been utilized by several previous studies in different academic domains [47,48,49,50,51,52,53,54,55,56,57,58,59,60,61]. PLS-SEM is a two-stage analysis technique; in the first stage, the measurement model is investigated for validity and reliability [62], while hypothesis testing is performed in the second stage, known as the structural model [63].

“Personal Construction Theory” was applied to record the perception of employees according to their own work and life experience as “everyone has the freedom to choose, the meanings one likes”. According to an earlier study by Chen, Lawler and Bae, “Asian participants generally select the central option while filling the questionnaires”. For the sake of avoiding this trend of central tendency bias, a six-point Likert scale ranging from strongly agree (1) to strongly disagree (6) was used. The scale’s alpha reliability was found to be considerable (EE = 0.79, JS = 0.81, OC = 0.71, SS = 0.75 and POS = 0.81).

## 3. Results

### 3.1. Measurement Model Evaluation

The first step in PLS-SEM analysis is the evaluation of the structural model for validity and reliability. For this purpose, the value of factor loadings and Composite Reliability (CR) should be above 0.70. At the same time, the values for Average Variance Extracted (AVE) should be above 0.50. The results in Table 1 show that all the above-stated values were achieved. Further, in order to test the Discriminant Validity (DV), we used the Heterotrait Monotrait (HTMT) ratio. The results in Table 2 show that the HTMT ratios were well below the threshold value of 0.85.

### 3.2. Structural Model Evaluation

The second step in the PLS-SEM evaluation is the assessment of the structural model. Bootstrap function with 5000 replicates was used to test the hypothesis.

To test the directional relationship between Extent of Exposure (EE)—Job Stress (JS) and Extent of Exposure (EE)—Organizational Commitment (OC), the analysis results are grouped in Table 3 as “Hypothesis Results by Bootstrapping”. As expected, it was estimated that the coefficients were found positive and statistically significant for the first proposition (EE→JS = 0.302, t-statistics = 3.631) which yielded enough evidence to accept hypothesis 1 where it was observed that a one-unit positive change in Extent of Exposure (EE) generates 0.302 units positive change in Job Stress (JS). Further to this, EE→OC = −0.208, t-statistics = 2.127 and JS →OC = −0.211, t-statistics = 2.961) which showed that a one-unit positive change in Extent of Exposure (EE) can introduce a negative 0.208-unit change in Organizational Commitment (OC) and vice versa. These results were found in accordance with the earlier studies [11,15,19,22]. These results enabled the acceptance of hypothesis 2. Further to this, it was also found that a one-unit positive change in Job Stress (JS) can produce negative change of 0.211 units in Organizational Commitment (OC) and vice versa. Again, as was expected, results convinced the acceptance of hypothesis 3.

### 3.3. Mediation Analysis

According to the hypothesis, four results are grouped in Table 4. With an aim to test the mediation effect of Job Stress (JS) in the relationship between Extent of Exposure (EE) and Organizational Commitment (OC), the present research first analyzed the significance of the indirect effect by employing a bootstrapping function of Smart PLS as recommended by a number of researchers [62,64]. Bootstrapping does not make any assumption regarding variable distribution shape or sampling distribution of the statistics. In addition to this, the effectiveness of bootstrapping is well established even for a small sample size. The indirect effect of Extent of Exposure (EE) on Organizational Commitment (OC) through Job Stress (JS) was significant (Beta = 0.159, t value = 3.63). As such, researchers [63] suggested that if both indirect, as well as direct, effects are significant while pointing in the same direction, then there would be complementary mediation; thus, Job Stress (JS) in the present study has a complementary mediating effect on the relationship between Extent of Exposure (EE) and Organizational Commitment (OC). These results are in accordance with the earlier studies [25,26]; this evidence prompted the acceptance of hypothesis 4.

### 3.4. Moderation Analysis

To test the moderating role of Perceied Organizational Support (POS) and Social Support (SS), this study utilized the product indicator approach. As such, hypothesis 5 predicted that Perceived Organizational Support (POS) moderates the relationship between Extent of Exposure (EE) to COVID-19 patients and Job Stress (JS). Further to this, the relationship was postulated as stronger with Low Perceived Organizational Support (LPOS) and weaker with High Perceived Organizational Support (HPOS) and Hypothesis 6 predicted that Social Support (SS) moderates the relationship between Extent of Exposure (EE) to COVID-19 patients and Job Stress (JS). Further to this, the relationship was stronger with Low Perceived Organizational Support (LPOS) and weaker with High Perceived Organizational Support (HPOS); so, results in Table 5 and Table 6 show that the POS significantly moderates the relationship between EE and JS, while results found no moderation impact on the relationship between EE and JS. It was also found that the relationship between Extnent of Exposure (EE) and Job Stress (JS) was strongest with LPOS and weaker when HPOS. The results presented in Table 5 show that there is a significant difference of HPOS and LPOS on the relationships between Extent of Exposure (EE) and Job Stress (JS). Hence, hypothesis 5 is supported while High Social Support (HSS) and Low Social Support (LSS) were not found to be statistically different. These results are in accordance with the earlier studies [27,29,31], thus rendered unsupportive to hypothesis 6.

## 4. Discussion

This study found that perceived organizational support moderated the relationship between the extent of exposure and stress, the relationship appearing weaker when organizational support was perceived as high and the relationship being depicted as stronger when with a low level of perceived organizational support. Further to this, it was also observed that Social support does not have a moderating impact on the relationship between the extent of exposure of doctors with COVID-19 patients and the job stress they feel in the workplace. Both relationships pointed out a significant reality that doctors are looking for support from their hospitals or government arrangements while at the same time they have almost no impact from the social side as they know that COVID-19 has become an illusion for the public. The public needs their support and listens to them carefully; they do not take any pressure from the public comments.

To the extent that these findings are replicated in future research, researchers suggest that one way to alleviate some of the deleterious effects of caring for COVID-19 patients may be to ensure that doctors have adequate support. Importantly, future research should focus on clarifying the mechanisms through which both types of support operate and the functions they serve. Moreover, efforts also should focus on other types of support that may be influential in this regard, such as the support provided by a cohesive work group or by special arrangements by the government. Although managers of health care organizations or hospitals may consider social support an issue unrelated to work, and one over which they have little control, organizational support is certainly under their influence. Additional research is needed to increase understanding of the stress-buffering effects of organizational support, the most effective means of providing and demon starting organizational support, and its likely antecedents. In terms of the latter, Eisenberger and colleagues [27] suggested that material and symbolic rewards may favorably affect perceptions of organizational support to the extent that they signify positive evaluations. Exposure to COVID-19 patients is a somewhat unique stressor in that COVID-19 carries an unknown mystery and has been accompanied by intensely stressed reactions in the public at large. Given the dramatic increase expected in the number of individuals infected with COVID-19, it is important that the managers of health care organizations monitor the effects of caring for COVID-19 patients on their employees’ psychological well-being as well as on their ability to be effective in their work roles while retaining sensitivity and compassion for the patients they care for. As was already mentioned, it is crucial that health care organizations do whatever they can to lessen the COVID-19-related distress of health care workers, specifically doctors.

To address the secondary mental health problems involved in the COVID-19 pandemic, the Emergency Psychological Crisis Intervention (PCIM) model could be developed and implemented through internet technology. Additionally, a detailed psychological crisis intervention plan should be developed, such as establishing a mental health intervention medical team, providing online courses to understand the psychological impact of stressful events, guiding medical personnel, and providing a hotline intervention of psychological assistance for medical treatment whereby the staff can discuss their psychological problems with well-trained professional mental health professionals. In this sense, the hospital must provide adequate support, including frequent shift systems, guarantee food and daily needs, and provide pre-employment training to resolve the identification and response to psychological problems of patients, families and themselves. In addition, the counselor or counselors should visit the doctor regularly to hear their experience and provide support so that the doctor can continue to maintain their commitment to the work, the hospital and the government of Pakistan.

Unfortunately, Pakistan is located between two main centers of corona virus, China and Iran, which were initially declared as the point of the outbreak of COVID-19, and there is a lot of traffic from these countries. Compared to China and Iran, Pakistan has a lower level of medical care and facilities, while the government is weaker. Due to its social and political structure, the Pakistani government will not be able to take measures against COVID-19, such as those shown by China and Iran. COVID-19 was discovered to be new to the world; it affects rich and poor countries alike. For various reasons, developing countries appear to be more vulnerable than developed countries. On the basis of the above factors, developing countries, like Pakistan, need to pay specific attention towards the psychological health of the doctors, so that doctors can work peacefully and share their learning with other doctors and staff members and remain helpful in stopping the spread of job stress.

This study is not without limitations. For example, because the data are cross-sectional, we cannot unambiguously determine the direction of causality. It may be that doctors’ job stress overestimates the extent of their exposure to COVID-19 patients. Another limitation is that data were self-reported. Several factors partially lessen this potential problem. First, this study measured the extent of exposure with objectively worded questions that did not frame exposure positively or negatively. Second, most theories of stress posit that an individual’s perception of a potential stressor is key to understanding the extent to which distress is experienced. Third, three of the hypotheses are concerned with interaction effects, not main effects.

## 5. Conclusions

The results of this study suggest that caring for COVID-19 patients may have an adverse effect on the psychological well-being of the health of doctors. More specifically, the extent to which doctors were exposed to COVID-19 patients as part of their role was significantly and positively associated with job stress at work. Although the extent of exposure was significantly and positively related to job stress, it was virtually unrelated to regular work, alternatively, as was expected. Since caring for COVID-19 patients was found to have been associated with job stress, and the prevalence of these patients is on the rise, it is important to identify alleviators of COVID-19-related distress. Both alleviators considered in this study, perceived organizational support and social support, touch on, to some degree, the extent to which the individual thinks he/she is valued and cared about, and lets others seek help from those who will help when necessary. The fact that distress was related to the extent of exposure partially mitigates this potential limitation. If job stress states were determined by the extent of exposure, it would be expected that doctors would be unable to perform their work as is required and the same distress may be transmitted to the other health-related staff. This concern also has little bearing on the hypothesized and detected moderating effects of perceived organizational support. Although organizational support was found to moderate the relationship between the extent of exposure and job stress significantly, the amount of variance the interaction effects accounted for was relatively small; their size suggests the need to investigate other potential moderators of this relationship.

This study marks the initial launch of the guidelines to provide diverse mental health dynamics and psychological interventions for Pakistani medical workers. The most advisable part of this study is a guideline to hospitals to pay more attention to the perception of their support towards the well-being of their doctors in the current working environment. Even in the presence of certain limitations, it is expected that this study will stimulate further empirical research on the effects of treating and handling COVID-19 patients on the psychological well-being of medical workers.

## Figures and Tables

**Table 1:** Assessment results (Factor Loading, Composite Reliability (CR) and Average Variance Extracted (AVE)).

Construct	Items	Loadings	CR	AVE
**Extent of Exposure (EE)**			0.839	0.636
	EE1	0.723		
	EE2	0.781		
	EE3	0.881		
**Job Stress (JS)**			0.905	0.706
	JS1	0.779		
	JS2	0.891		
	JS3	0.897		
	JS4	0.786		
**Perceived Organizational Support (POS)**			0.911	0.633
	POS1	0.891		
	POS2	0.814		
	POS3	0.700		
	POS4	0.813		
	POS5	0.784		
	POS6	0.758		
**Social Support (SS)**			0.890	0.574
	SS1	0.742		
	SS2	0.719		
	SS3	0.773		
	SS4	0.701		
	SS5	0.819		
	SS6	0.785		
**Organizational Commitment (OC)**			0.885	0.563
	OC1	0.722		
	OC2	0.739		
	OC3	0.739		
	OC4	0.789		
	OC5	0.767		
	OC6	0.744		

**Table 2:** Discriminant validity (by Heterotrait Monotrait—HTMT ratio).

Factors	HTMT Ratio
**Extent of Exposure (EE)**	0.511
**Job Stress (JS)**	0.325
**Perceived Organizational Support (POS)**	0.441
**Social Support (SS)**	0.431
**Organizational Commitment (OC)**	0.321

**Table 3:** Hypothesis results by bootstrapping (direct relationships).

Hypothesis	Path	Path Coefficient	t Statistics (|O/STDEV|)	*p* Values
**H1**	EE->JS	0.302	3.631	0.000
**H2**	EE->OC	−0.208	2.127	0.000
**H3**	JS-OC	−0.211	2.961	0.000

**Table 4:** Mediation analysis (indirect relationship).

Hypothesis	Indirect Path	Path Coefficient	T Statistics (|O/STDEV|)	*p* Values
**H2**	EE ->JS -> OC	0.159	3.63	0

**Table 5:** Results of moderation Analysis of Perceived Organizational Support on Extent of Exposure (EE)–Job Stress (JS).

Relationship	β Values	t-Value	*p*-Values	Result
**EE->JS**	0.135	2.02	0.00	Supported

**Table 6:** Results of moderation analysis of SS on the relationships between EE–JS.

Relationship	β Values	T-Value	*p*-Values	Result
**EE->JS**	0.060	0.201	0.218	No moderation
